# Rare syringoid eccrine carcinoma of the upper lip and nasal base treated with resection and subsequent innovative reconstruction using an Abbé flap, turbinate flaps and three-stage forehead flap: a case report

**DOI:** 10.1186/s12957-022-02754-4

**Published:** 2022-09-08

**Authors:** Zdeněk Dvořák, Richard Pink, Petr Heinz, Jaroslav Michálek, Petr Krsička

**Affiliations:** 1 Department of Plastic and Aesthetic Surgery, St. Anne’s Faculty Hospital and Medical Faculty of Masaryk University, Berkova 34, 612 00 Brno, Czech Republic; 2 Department of Oral and Maxillofacial Surgery, University Hospital and Medical Faculty of Palacky University, I. P. Pavlova 6, 77900 Olomouc, Czech Republic; 3 Department of Clinical and Molecular Pathology, University Hospital and Medical Faculty of Palacky University, I. P. Pavlova 6, 77900 Olomouc, Czech Republic; 4 Department of Surgical Oncology, Masaryk Memorial Cancer Institute and Medical Faculty of Masaryk University, Zluty Kopec, 7, 656 53 Brno, Czech Republic

**Keywords:** Syringoid eccrine carcinoma, Head and neck malignancy, Nasal reconstruction, Turbinate flap, Functional endonasal surgery

## Abstract

**Background:**

Although syringoma is a common benign tumour of the sudoriferous gland, there is also an extremely rare malignant form known as syringoid eccrine carcinoma (SEC). SEC usually exhibits slow growth with deep invasion and a frequent tendency to relapse. The treatment of choice is radical wide resection, which poses a difficult reconstructive problem, especially when the tumour is located in the centre of the face.

**Case presentation:**

In this case, a 70-year-old man was diagnosed with an SEC at the same location as a benign syringoma of the upper lip and nasal base that had undergone primary excision 7 years prior. Primary radical resection was performed with immediate Abbé flap reconstruction. Nevertheless, histology revealed positive margins, and 3 additional re-excisions were needed to achieve clear margins. Four months after the initial resection, the patient had undergone an innovative reconstruction technique including not only the Abbé flap but also a turbinate flap harvested with functional endonasal surgery and a three-stage forehead flap.

**Conclusion:**

To the best of our knowledge, this is the first case report of a suspect malignant transformation of a benign syringoma after 7 years. In addition, from oncoplastic and reconstructive points of view, the bilateral use of the turbinate flap for reconstructing the intranasal lining of the alar base is unusual, and the use of functional endonasal surgery in nasal reconstruction for reducing the risk of damaging the vascular supply of the flap is innovative.

## Introduction

Primary eccrine tumours form a very diverse group of benign and malignant tumours with a broad histological spectrum, which probably creates inconsistencies in the classification and terminology of these tumours [1–4].

Syringoma is a common benign tumour originating from the eccrine ducts. It most often occurs in middle-aged women in the area around the eyelids; however, various other clinical variants and localisations have also been described [5]. A very rare malignant form of syringoma was first described by Freeman and Winkelmann as a basal cell carcinoma with eccrine differentiation (eccrine epithelioma) in 1969 [6]. Mehregan divided eccrine epithelioma (adenocarcinomas) into 4 basic variants: porocarcinoma, syringoid eccrine carcinoma (SEC), mucinous eccrine carcinoma and clear cell eccrine carcinoma. SECs, accounting for less than 0.01% of skin malignancies, are known by many other names, such as syringomatous carcinoma, malignant syringoma, eccrine gland adenocarcinoma, squamous eccrine ductal carcinoma and sclerosing sweat ductal carcinoma [1, 7]. These tumours exhibit slow growth and deep invasion and thus has a frequent tendency to relapse; however, it does not usually metastasise [8]. Sidiropoulos et al. identified over 40 SEC cases worldwide in the literature in 2011 [1].

The clinical manifestation lacks a uniform image, but most lesions are nodular or planar with a size of over 1 cm on the head or neck (ear, nose, upper lip, eyelid, scalp, cheek) and less often on the trunk or extremities [7]. SEC is a slow but markedly locally aggressive growth [1] with deep and perineural invasion, so it often relapses.

In the management of SEC, no standard of care has been established, but wide surgical excision has been effective in 70–80% of cases [9]. However, if the tumour is located in the centre of the face, a complex reconstructive situation arises due to the disruption of the airway and the compliance of the oral cavity.

Modern principles of nasal reconstruction are based on several basic principles, mostly established or confirmed by Millard, Burget and Menick at the end of the twentieth century [10–12]. The oldest method is to choose the forehead flap as the best donor for nasal skin reconstruction, preferably with a three-stage approach. Comprehensive reconstruction of the supportive component is a necessary and integral part of nasal reconstruction to ensure the stability of the reconstruction result over time. In order to follow the above principles, it is necessary to reconstruct the nose or parts of it on an intranasal lining with a good vascular supply [10, 13, 14].

## Case presentation

**Fig. 1:**
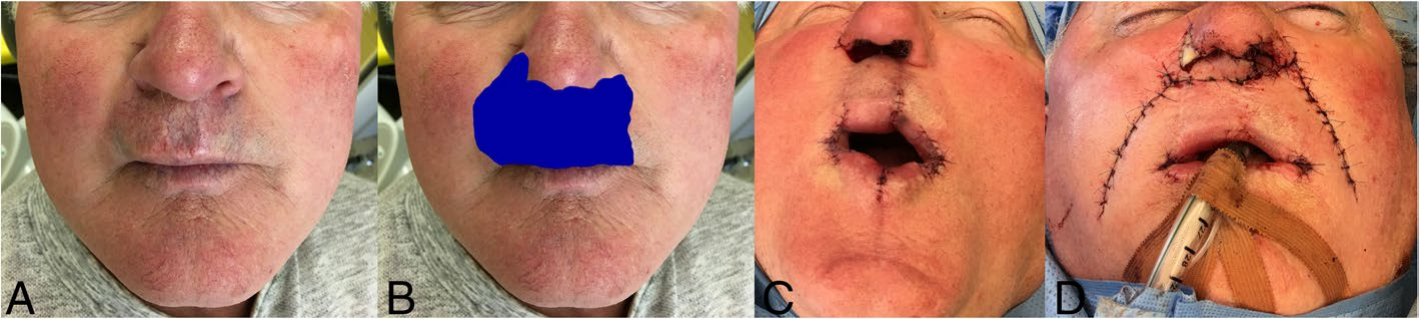
Condition of the patient before resection and primary reconstruction. **A** Appearance of the patient before resection. **B** Actual extent of total resection. **C** Condition at the end of the 3rd operation. **D** Condition at the end of primary lip and nose reconstruction

The patient was a 70-year-old retired Caucasian man who was originally treated for swelling of the base of the upper lip with a small palpable lesion and upper incisor toothache at the Department of Oral and Maxillofacial Surgery in 2010 (Fig. 1). The patient’s past medical history was significant for hypertension and cardiac arrhythmia (asymptomatic detection of idiopathic nonsinus ventricular tachycardia), and he was undergoing treatment with a beta-blocker. He did not report other diseases and was a nonsmoker. At that time, amoxicillin/clavulanic acid 500/125mg was prescribed due to a suspected infection. However, his condition did not improve at all. Diagnostic excision was performed, and typical structures of syringoma were found in the histopathological findings: well-circumscribed proliferation with epithelial cells forming ductules, nests and cords. Cells were basaloid and cuboidal and some of them with clear cytoplasm and double layered (in ductules), ducts were often comma or tadpole-shaped or in a paisley pattern. There were no cytologic atypia and no mitoses. The tumour was located in the superficial reticular dermis without infiltrative growth into the skeletal muscle at the bottom of the excision. Due to the fragmentation of the material, it was not possible to reliably comment on the completeness of the excision, with the comment that recurrence is possible.

There was no further adjuvant therapy, and the patient underwent standard follow-up observation by his general practitioner.

After 7 years, the patient was referred to our hospital again, presenting with upper lip swelling and a palpable upper lip lesion measuring 2 cm × 3 cm with infiltration of the fornix vestibuli and columellar base. The lesion was greyish in colour with telangiectasias, and it was painless and hard in consistency. No lymphadenopathy of the cervical lymph nodes was observed. There was no family history of any cancer.

**Fig. 2:**
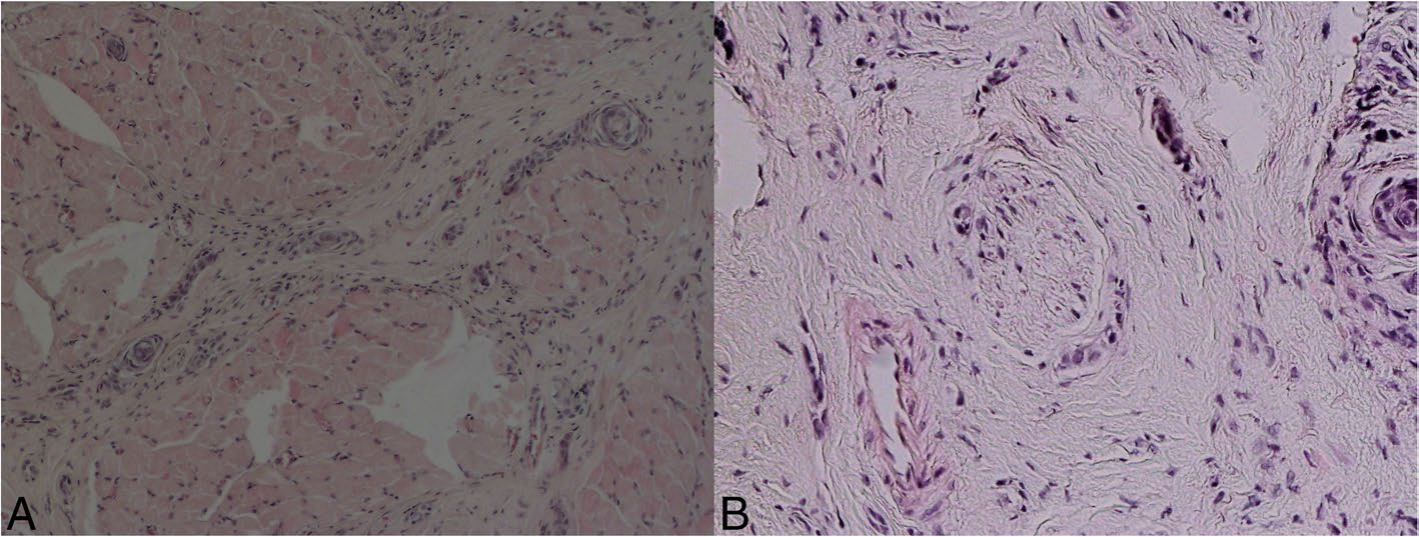
**A** Histological image of SEC showing infiltrative growth between the skeletal muscle fibres; haematoxylin-eosin staining, magnification × 100. **B** SEC with perineural spread; haematoxylin-eosin staining, magnification × 200

An assessment was performed by orthopantomography (OPG), followed by a diagnostic biopsy. Histology revealed significantly enlarged submucosal stroma that was captured and infiltrated by small, predominantly solid cell pins with a hint of luminal formation or tadpole configurations in some places. The cells were medium in size and cytologically uniform, with vesicular nuclei, small nucleoli and hyperchromic foci. Mitosis was not detected. The tumour extended between the fibres of the skeletal muscle, and perineural propagation was also evident. No necrosis was present. Cytologic features were similar to the benign counterpart (syringoma); however, there were some malignant signs that distinguished the SEC from the syringoma, in particular, infiltrative growth and perineural invasion. Immunohistochemically, the cells were positive for cytokeratin AE 1/3, CK5/6 and p63, as well as CK7 very rarely and p53 weakly and focally. The cells were negative for CK20, epithelial cell adhesion molecule (Ber-EP4), B cell lymphoma 2 protein (Bcl2), smooth muscle actin (SMA), S-100, epithelial membrane antigen (EMA), carcinoembryonic antigen (CEA), oestrogen receptor (ER) and progesterone receptor (PR). The Ki67 proliferation index was up to 5%. Histologically, the findings indicated an adnexal tumour, most likely SEC, with a probability of over 50% (Fig. 2).

The chest X-ray results were also normal. Computed tomography (CT) showed a small hypodense soft tissue mass in the upper lip. In addition, there was no evidence of bone invasion or destruction.

Staging positron-emission tomography (PET)/CT showed no distant metastases.

Radical resection was planned with immediate reconstruction of the upper lip from the lower lip using an Abbé flap. The nasal base and septum were also included in the resection. Assessment of the intraoperative frozen sections of the mass revealed negative margins. According to the definitive histology of the resected tissue, however, the tumour reached the edges of the excision area in the left nasal entrance, and close margins were found bilaterally on the lip. the second, more radical resection of the nasal base was performed under general anaesthesia, including half of the columella and both lip margins. The frozen section again showed negative margins; but similar to the previous procedure, according to the definitive histology, a close, positive resection margin of 1 mm was found at the right nasal ala. In the third operation under local anaesthesia, the pedicle of the Abbé flap was disconnected, and the right nasal ala was re-excised. Bilateral commissuroplasty was also performed for contracture of the oral opening, also described as microstomia. The Fairbanks and Dingman technique was used for the vermilion [15], and the muscle was split into two layers by crossing the surface layer, according to the technique described by Villorio [16]. As histology again revealed a close, positive margin on the right nasal ala, re-excision was performed up to half the height of the nostril. Finally, histology of the newly excised tissue showed no tumour cells, and only reparative changes were observed.

After 4 months without recurrence, the nose base was reconstructed with two nasolabial flaps reinforced by the implantation of a double cartilaginous graft from the rest of the nasal septum. The defect of the right nasal ala was adjusted with a composite graft containing both skin and subcutaneous tissue with cartilage from the helix of the left ear. Unfortunately, during the postoperative period, necrosis developed in the caudal half of the composite graft, and over the next 10 months, the necrosis affected half of the right nasal ala, the patency of the right nostril became limited, the colloid and tip of the nose collapsed and mild microstomia persisted. Therefore, a team of plastic and maxillofacial surgeons decided that a new, complex, three-stage nasal reconstruction procedure would be performed, including adequate reconstruction of the intranasal lining and improvement of the nasal support system to withstand severe scarring.

**Fig. 3:**
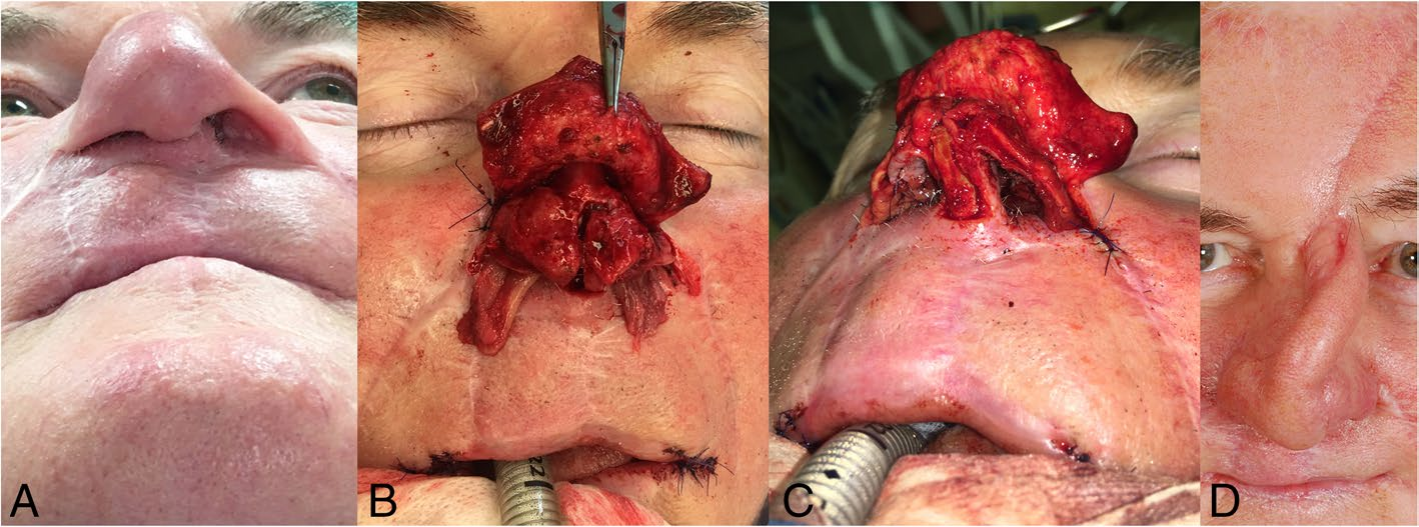
Secondary nose reconstruction. **A** Condition before surgery. **B** Unfolding of the collapsed and constricted nostrils with an elevation of the bilateral turbinate flaps (caudally extending from the nostrils). **C** Nose skeleton reconstruction. **D** Condition 6 weeks after the secondary reconstruction of the nose before thinning and detachment of the forehead flap

**Fig. 4:**
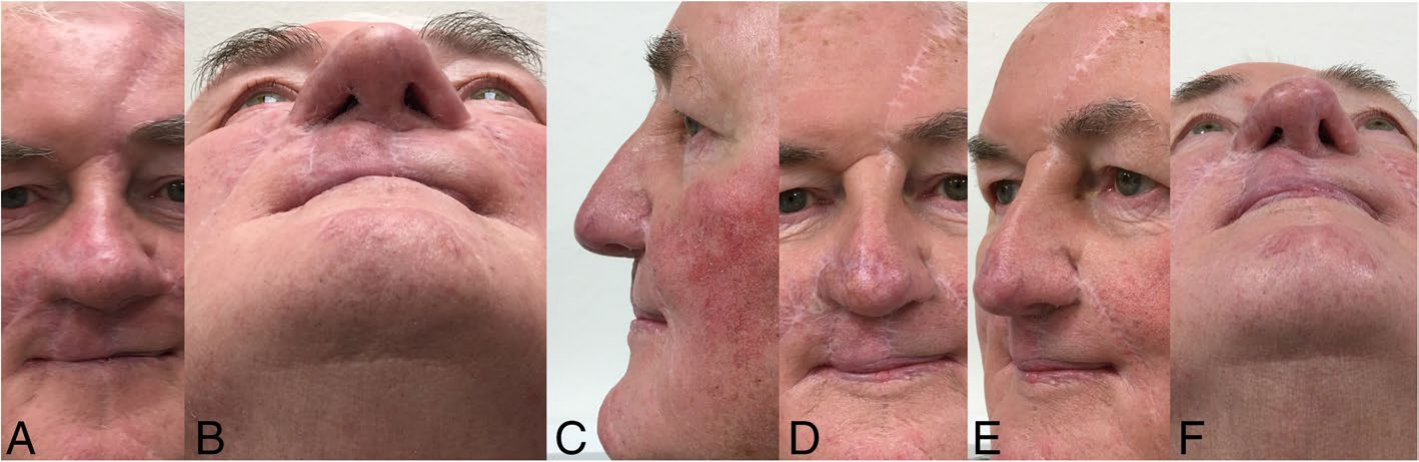
**A**, **B** Final condition 2 months after the secondary nose reconstruction. **C**–**F** Final condition 50 months after the primary operation and 23 months after the last operation

Seventeen months after the first operation and 14 months after the complete resection of the tumour without recurrence, the nasal reconstruction procedure was performed in parallel with another commissuroplasty. After the skin of the nose was elevated, the nasal skeleton was exposed and returned to its original dimensions. Simultaneously, the cartilage was taken from the right sixth rib and the cavum conchae to reconstruct the nasal septum and nasal alae, respectively. For reconstruction of the missing intranasal lining of the lower half of the nostrils, the turbinate flap was selected. Each turbinate flap was obtained by functional endonasal surgery (FES) and sewn into the defect of the intranasal lining. Then, the skeleton of the nose was completed, a central pillar from the costal cartilage was constructed to support the newly created nasal ala, and the nasal alae were reinforced with the use of the (harvested) conchal cartilage. The missing skin cover of the right nasal ala and the front of the columella was replaced using a left paramedian forehead flap. Recovery lasted 6 weeks (Fig. 3D). In phase II of the nose reconstruction, the new skin cover was thinned, and in phase III, a month later, the final disconnection of the pedicle was performed. Over the next 8 months, both nasal entrances were widened with Z-plasty in the area of the soft triangle, Z-plasty was performed to advance the right nostril and VY-plasty was performed to advance the left nostril (Fig. 4). The individual treatment steps are summarised in Table 1.

**Table 1:** Chronological overview of individual surgical steps (*m* months, *LA* local anaesthesia, *GA* general anaesthesia)

Operation number	Time scale	Operational performance	Complication
1	0	Resection of the tumour of the upper lip and nasal base with immediate reconstruction of the upper lip according to Abbé under GA	In the left nasal entrance, the tumour extended into the excision area; bilateral tight margins at the lip.
2	1 m	Resection of the nasal base and both margins of the upper lip under GA	Close resection margin, 1 mm at the right nasal wing.
3	2 m	Detachment of the pedicle of the Abbé flap from the lower lip, re-excision of the tumour in the area of the right ala, bilateral commissuroplasty under LA	Residual structures of syringoma up to 1 mm at the right nasal ala.
4	3 m	Re-excision of tumour margins on the right ala to half the height of the wing under LA	No residual tumour structures on histology.
5	7 m	Primary reconstruction of the nose with two nasolabial flaps, reinforcement of the columella with a septal graft and reconstruction of the right nasal ala with a composite auricular graft under GA	Loss of the composite graft of the right ala, limited patency of the right nostril, collapse of the columella and the tip of the nose, microstoma.
6	17 m	Commissuroplasty, elevation of both turbinate flaps from the inferior conchae, cartilage graft harvesting from both auricles and from the 6th right rib, reconstruction of the nasal framework and covering with the left paramedian forehead flap under GA	Prolonged healing, repeated capillary bleeding from the right nostril.
7	18 m	Re-elevation and thinning of the forehead flap under LA	
8	20 m	Removal of the supply pedicle from the forehead under LA	
9	27 m	Enlargement of both nostrils by Z-plasty in a soft triangle, Z-plasty of the right alar attachment and V-Y advancement of the left nasal ala under LA	

During a follow-up and clinical examination, an ultrasound of the cervical nodes with subsequent PET/CT did not show any regional or distant metastases. The patient has remained recurrence-free now for 60 months after the tumour resection. The patient was satisfied with the aesthetic result of the reconstruction.

## Discussion

This case report is exceptional for the following reasons. Firstly, histopathologically, there is no other mention of the suspect malignant transformation of a benign syringoma 7 years after excision in the available medical literature, and SEC is still extremely rarely described. In 2015, Frouin et al. reported 30 French patients with eccrine carcinoma from 1995 to 2008. In their cohort, the immunohistopathology of only 5 patients was SEC [17]. Similarly, in 2001, Urso et al. reported a cohort of 60 patients with eccrine carcinoma, and further assessment revealed SEC in only 3 patients [3]. Secondly, from a reconstructive point of view, the use of bilateral turbinate flaps for reconstruction of the intranasal lining of the alar base and the safe harvesting of this flap using FES, minimising the risk of damage to the vascular supply, is unique.

The main method of treatment for localised lesions is radical surgical excision with negative margins. Therefore, Mohs’s micrographic surgery is the method of choice for the treatment of this tumour, and it is preferred over controlled excision. In the case of a metastatic process, both chemotherapy and radiotherapy have been described in the literature [1]. Unfortunately, in our case, Mohs’s micrographic surgery was unavailable, and over the course of 3 consecutive excisions, a deep invasion of the SEC was discovered. For that reason, significantly more extensive resection was needed than originally expected; the extent of resection was not reached during the immediate reconstruction, which forced the implementation of double commissuroplasty bilaterally. Maintaining the postoperative result is challenging, as there is often local tissue scarring and subsequent recurrence [1, 9, 10].

The failure of the initial nasal reconstruction can be attributed to the unreliability of the composite graft in the alar area, especially when its size approached the referenced maximum adherent size of 1 cm for a composite graft [10]. The second reason was the insufficient size and mechanical resistance of the cartilaginous graft from the rest of the septum, which was not able to maintain the nasal tip projection during the extensive postoperative healing and scarring. Although the application of nasolabial flaps for reconstruction of the nasal base of the nose and columella was successful [10, 11], there was a problem with nasal patency caused by too bulky soft tissues of the flap. Every other flap used in the reconstruction—local, pedicled or free flap—obturates the nostrils. The internal layer must be thin, pliable and very well vascularised to nourish the nasal framework that is built upon it. The best tissue for an internal lining of a reconstructed nose is nasal mucosa, usually harvested from the nasal septum [10], but this tissue source was consumed during resection. Thus, a revision nasal reconstruction operation was performed for this case. The thin alar lining was obtained using bilateral turbinate flaps. The turbinate flap is obtained from the mucosa of the lower nasal concha, which can be harvested with a ventral supply from the ethmoidal anterior artery and alar nasal artery, a branch of the facial artery, or, alternatively, a dorsal supply from the descending branch of the sphenopalatine artery [18]. This flap was originally described for use in septal perforation closure [19, 20], palatal fistula closure [21] and cranial base defect repair [22]. In 1999, Mukarami published a paper in which he described the cases of 9 patients from a cohort of 18 in whom he used a turbinate flap to reconstruct the intranasal lining during the reconstruction of small full-thickness defects of the nasal ala or wall. Additionally, in his small cadaveric study, he defined the dimensions of the flap that can be used—usually 5 cm^2^, with a flap length of 2.8 cm (1.7–4.0 cm) and a width of 1.7 cm (1.5–2.0 cm) [23]. We harvested two flaps—1.7 × 3 cm and 2 × 4 cm—in their maximum size, using FES, which allowed us not to lose any part of the harvested flaps.

The cartilage from the 6th right rib was used as a strut graft for the columella, as it provides sufficient strength and is available in sufficient quantity to reconstruct the support for the tip of the nose [24]. Conchal cartilage was used to reconstruct the alar cartilage and support the margins of both nasal alae. Only a complete reconstruction of the supporting layer of the nose ensures the stability of the result over time [10, 12, 13]. The skin defect was repaired by a three-stage forehead flap, which has been shown to be the most suitable solution for repairing the larger skin defects on the caudal part of the nose [14]. The patient is now 5 years post-reconstruction with a stable reconstructive result.

## Conclusion

In our case, the tumours’ histopathological findings show that malignant transformation of a benign syringoma in SEC is a likely possibility. In patients with SEC, it is critical to achieve negative margins with controlled excision, after which reconstruction can be performed. For reconstruction of large complex defects of the nasal base, the turbinate flap harvested by FES represents alternative thin and pliable tissue suitable for the reconstruction.

## Data Availability

The data are not publicly available due to the data being confidential patient data but are available from the corresponding author on reasonable request.
